# Spatial-temporal evolution and convergence analysis of agricultural green total factor productivity—evidence from the Yangtze River Delta Region of China

**DOI:** 10.1371/journal.pone.0271642

**Published:** 2023-03-20

**Authors:** Hongjie Bao, Xiaoqian Liu, Xiaoyong Xu, Ling Shan, Yongteng Ma, Xiaoshuang Qu, Xiangyu He

**Affiliations:** 1 School of Management, Northwest Minzu University, Lanzhou, China; 2 Research Institute of Economics and Management, Southwestern University of Finance and Economics, Chengdu, China; 3 Department of Logistics, LanZhou University, Lanzhou, China; 4 School of Business Administration, Zhongnan University of Economics and Law, Wuhan, China; 5 School of Economic, Northwest Minzu University, Lanzhou, China; 6 Business School, Zhengzhou University of Aeronautics, Zhengzhou, China; 7 Cantoese Merchants Business School, Guangdong University of Finance and Economics, Guangzhou, China; Central Queensland University, AUSTRALIA

## Abstract

Measuring regional differences in agricultural green total factor productivity (AGTFP) provides a basis for policy guidance on agricultural green development in the Yangtze River Delta (YRD) region. By constructing a two-period Malmquist–Luenberger index under the carbon emission constraint, we measure the AGTFP of cities in the YRD region from 2001 to 2019. Furthermore, adopting the Moran index method and the hot spot analysis method, this paper analyzes the global spatial correlation and local spatial correlation of AGTFP in this region. Moreover, we investigate its spatial convergence. The results show that the AGTFP of 41 cities in the YRD region is on an increasing trend; the growth of AGTFP in the eastern cities is mainly driven by green technical efficiency, while this growth in the southern cities is mainly stimulated by green technical efficiency and green technological progress. We also find a significant spatial correlation between cities’ AGTFP in the YRD region from 2001 to 2019, but with certain fluctuations, showing a U-shaped trend of "strong-weak-strong". In addition, absolute *β* convergence of the AGTFP exists in the YRD region, and this convergence speed is accelerated with the addition of spatial factors. This evidence provides support for implementing the regional integration development strategy and optimizing the regional agricultural spatial layout. Our findings offer implications for promoting the transfer of green agricultural technology to the southwest of the YRD region, strengthening the construction of agricultural economic belts and agricultural economic circles, and improving the efficiency of agricultural resource use.

## 1. Introduction

Ecological priority and green development are vital goals of China’s high-quality development [1–4], and agricultural green development is one of its crucial components [5–7]. However, in the past, to maximize agricultural output, China put vast pesticides and chemical fertilizers into agricultural production, which not only caused serious pollution of land, water bodies, and agricultural products [8–10], but also increased greenhouse gas emissions [11], leading to climate change and the destruction of ecosystems and posing a serious threat to human survival [12–14]. Agriculture, as an essential source of greenhouse gases, has attracted widespread social concern [15]. Therefore, China’s agricultural development should lay emphasis on green transformational development [16].

The YRD region plays a leading role in China’s economic development, covering three provinces (Anhui, Zhejiang, and Jiangsu) and one municipality (Shanghai). Statistics show that in 2020, the YRD region, with less than 4% of China’s land area, generated nearly 1/4 of China’s total economic growth and 1/3 of its total imports and exports. Therefore, it plays a vital role in China’s transformation and upgrading. Meanwhile, the YRD region has an excellent agricultural foundation and rich ecological resources. Therefore, agricultural green development of the YRD region is crucial to realize the transformation and upgrading of agriculture, and it is also an important part for this region to construct a high-level ecological green integration demonstration area.

Total factor productivity (TFP), especially green total factor productivity (GTFP), considering resource and environmental pollution constraints, is a critical indicator to measure economic green development [17–19]. This indicator is currently used to study the industrial sector [10,20,21], while research on the agricultural green total factor productivity (AGTFP) is less frequent [22]. The AGTFP is an indicator for assessing agricultural growth based on the framework of the neoclassical development model, which is favored by numerous scholars. The existing literature on AGTFP has mainly explored from its measurement and influencing factors. A large body of literature has used provincial-level data to study AGTFP. Extant studies on measuring AGTFP mainly adopt two methods: the stochastic frontier analysis [23–25] and the data envelopment analysis [26–29]. The stochastic frontier analysis (SFA) is a typical representative of the parametric approach in frontier analysis, which requires setting a specific production function and a compound perturbation term, while the frontier production function is susceptible to the influence of the individual region [30]. The data envelopment analysis (DEA) is a non-parametric approach, which does not require the setting of a specific functional form; it is a frontier production function applicable to multiple inputs and multiple outputs [31]. As SFA can only use a single output to measure efficiency; in other words, SFA cannot handle undesirable outputs. Therefore, DEA is the most widely used method to measure AGTFP [32]. Other scholars have focused on exploring the determinants of AGTFP. For instance, studies at the provincial level show that agricultural insurance [33,34], agricultural services [35], agricultural fiscal expenditure [36], land market distortions [37], and carbon trading [38] affect AGTFP. A few emerging studies have begun to study AGTFP from the city level, and the limited literature reveals that low-carbon pilot city policy [39] and economic agglomeration [40] have impacts on AGTFP.

Most studies have used provincial-level data to examine AGTFP, while there is far less evidence at the city level. The limited studies at the city level have focused on the investigation of its influencing factors, while systematic evidence on exploring the spatial correlation and regional synergy of AGTFP at the city level in China is still scarce.

The measurement of agricultural greenhouse gas emissions is a key aspect of studying agricultural pollution emissions [41,42]. Agricultural greenhouse gas emissions mainly come from livestock farming, crops, and various land-use types [43,44]. Data from the Intergovernmental Panel on Climate Change (IPCC) and the World Meteorological Organization (WMO) report that agricultural carbon emissions contribute more than 30% to total global carbon emissions [45]. Therefore, exploiting the coefficient method constructed by the IPCC, we measure agricultural greenhouse gases. Specifically, agricultural greenhouse gases mainly originate from animal husbandry, rice cultivation, agricultural materials, and straw burning, which produce not only CO_2_ emissions but also other greenhouse gas emissions, such as CH_4_, N_2_O, perfluorocarbons, HFCs, etc. Referring to existing studies [46,47], this paper converts these greenhouse gas emissions into CO_2_ emissions.

Previous studies have mainly examined China’s AGTFP at the provincial level [22,48], but evidence on regional AGTFP, especially in the YRD region, is still scarce. Specifically, under the construction of a high-level ecological green integration demonstration area, what are the current status and evolution characteristics of AGTFP in the YRD region? What factors constrain the AGTFP in this region? How does the AGTFP vary spatially between cities in the region? Exploring these questions is conducive to judging the status of AGTFP and accurately grasping the potential for sustainable agricultural development.

The potential innovations of this paper are as follows. First, we measure the AGTFP of cities in the YRD region. In contrast to a large body of literature that explores AGTFP by using provincial data, we systematically explore the spatial correlation and regional synergy (convergence) of AGTFP at the city level in China, which enriches the existing literature on urban AGTFP. Second, adopting global spatial correlation and local spatial correlation, we depict the spatial and temporal trends of AGTFP. Specifically, employing Moran’s I index to analyze the spatial autocorrelation of AGTFP, we attempt to accurately assess the effectiveness of the agricultural industry in the YRD region, which can fulfill the sustainable development goal that coordinates agricultural environmental protection with farmers’ income growth. Third, we introduce the spatial effect factor into the test of classical *β* convergence. Through the established spatial econometric model, we estimate its spatial convergence, discuss its divergent characteristics, and explore the spillover effect of AGTFP, which provides a basis for regional synergy to accelerate the agricultural green growth in the YRD region.

The rest of this study is structured as follows: Section 2 shows the methodology and data sources. Sections 3 and 4 present the empirical analysis and convergence analysis, respectively. Section 5 displays conclusions and recommendations.

## 2. Methodology and data sources

### 2.1. Measurement of the GTFP index

When calculating TFP by using parametric methods, a pre-defined production function is required. As the setting of the production function usually varies widely and needs to be supported by a large number of samples, the TFP measured based on this method usually has large differences. In contrast to the parametric method, the non-parametric method does not require setting a specific production function form, which can avoid the residual autocorrelation issue. Moreover, this method can decompose the TFP index, which is conducive to exploring the driving force of TFP growth. Therefore, we adopt the non-parametric method to measure the AGTFP in the YRD region. The directional distance function (DDF) encourages a producer to simultaneously increase the production of good output and cut down the production of bad outputs, which is consistent with the concept of sustainable development of production processes [49]. Based on DDF, Chung et al. proposed the Malmquist–Luenberger (ML) index to incorporate undesirable outputs, such as pollutant emissions, into the measure of GTFP [50].

The solution of the DDF can be obtained by the following equation.

$$\{\begin{array}{l} \sum_{0}^{t}(x^{t},y^{t},b^{t};g^{t})=\max\delta \\ s.t.\\ \sum_{m=1}^{M}w_{m}^{t}y_{mn}^{t}\geq (1+\delta )y_{mn}^{t},n=1,2,\cdots N;\\ \sum_{m=1}^{M}b_{ml}^{t}=(1-\delta )b_{ml}^{t},l=1,2,\cdots L;\\ \sum_{m=1}^{M}w_{m}^{t}x_{mp}^{t}\leq x_{mp}^{t},p=1,2,\cdots P;\\ w_{m}^{t}\geq 0;m=1,2,\cdots M \end{array}$$ (1)

where x, y, and b represent input factors, desirable outputs, and undesirable outputs, respectively. *m* refers to the *M* production decision units; *P*, *N*, and *L* are the types of input factors, desirable outputs, and undesirable outputs. *δ* denotes the value of DDF in period *t* with the requirement of maximizing desirable output y and minimizing undesirable output b. *w* is the weight, i.e., the share of each producer’s output in GDP [50].

The ML index is adopted to measure the AGTFP considering desirable outputs as well as undesirable outputs, such as agricultural output and pollutant emissions. The ML index is defined as:

$$ML_{t}^{t+1}={\left\{\frac{\left[1+D_{0}^{t}(x^{t},y^{t},b^{t};g^{t})\right]}{\left[1+D_{0}^{t}(x^{t+1},y^{t+1},b^{t+1};g^{t+1})\right]}\times \frac{\left[1+D_{0}^{t+1}(x^{t},y^{t},b^{t};g^{t})\right]}{\left[1+D_{0}^{t+1}(x^{t+1},y^{t+1},b^{t+1};g^{t+1})\right]}\right\}}^{\frac{1}{2}}$$ (2)

where $D_{0}^{t}(x^{t},y^{t},b^{t};g^{t})$ and $D_{0}^{t+1}(x^{t},y^{t},b^{t};g^{t})$ denote the distance functions in periods *t* and *t+1*; *g* = (*y*,−*b*) represents output-oriented. $D_{0}^{t}(x^{t+1},y^{t+1},b^{t+1};g^{t+1})$ and $D_{0}^{t+1}(x^{t+1},y^{t+1},b^{t+1};g^{t+1})$ refer to the mixed distance functions under technical conditions in periods t and t+1. The ML index depicts the change in TFP from period t to period t+1; a value greater than 1 indicates an increase in GTFP; a value less than 1 implies a decrease in GTFP.

To further explore the changes in AGTFP in the YRD region, we decompose the ML index into two components: efficiency change (EFFCH) and technical change (TECH). EFFCH indicates the output growth due to efficiency changes within agriculture, which is mainly driven by the changes in pure efficiency and production scale, while TECH indicates the output growth propelled by technological progress, which are calculated by Eqs (3) and (4).


$$EFFCH_{t}^{t+1}=\frac{\left[1+D_{0}^{t+1}(x^{t},y^{t},b^{t};g^{t})\right]}{\left[1+D_{0}^{t+1}(x^{t+1},y^{t+1},b^{t+1};g^{t+1})\right]}$$ (3)

$$TECH_{t}^{t+1}={\left\{\frac{\left[1+D_{0}^{t+1}(x^{t},y^{t},b^{t};g^{t})\right]}{\left[1+D_{0}^{t}(x^{t},y^{t},b^{t};g^{t})\right]}\times \frac{\left[1+D_{0}^{t+1}(x^{t+1},y^{t+1},b^{t+1};g^{t+1})\right]}{\left[1+D_{0}^{t}(x^{t+1},y^{t+1},b^{t+1};g^{t+1})\right]}\right\}}^{\frac{1}{2}}$$ (4)

The TECH index measures the convergence of each producer to the optimal production frontier from period t to t+1, representing the output growth caused by technological progress, i.e., the "growth effect". The EFFCH index measures each producer’s catch-up on the production possibility frontier from period t to period t+1, indicating the output growth induced by internal efficiency changes, i.e., the "catch-up effect". EFFCH > 1 and TECH > 1 indicate technical efficiency improvement and frontier technology progress, respectively, and EFFCH < 1 and TECH < 1 mean technical efficiency deterioration and frontier technology regression, respectively.

We selected input–output variables considering the reasonableness and availability of data. For input variables, based on the "five factors theory" of agricultural production, input factors include labor, land, capital, water resources, and electric energy, which are necessary for agricultural development. Therefore, we use the number of laborers employed in agriculture, forestry, and fisheries to measure labor input and adopt the sum of crop sown area and aquaculture area to represent land input. Moreover, due to the existence of radial and non-radial relationships between agricultural inputs and outputs, we select fertilizer, machinery, pesticides, agricultural films, and diesel to measure capital inputs. For output variables, the desirable output variable is measured by the total output of agriculture, forestry, animal husbandry, and fishery and is adjusted by constant prices in 2000. Table 1 displays the variables that measure the AGTFP index.

**Table 1:** The measurement of the AGFTP index.

Indicator Categories	Variables		Evaluation indicator	Unit
Input indicators	Labor input		Number of people employed in agriculture	Population per million
	Land input		Crop acreage	Per thousand hectares
			Aquaculture area	Per thousand hectares
	Capital input		Total power of agricultural machinery	Per million kilowatts
			Agricultural fertilizer application	Ten thousand tons of
			The amount of organic fertilizer used	Ten thousand tons of
			The consumption of Chemical Pesticides	Ten thousand tons of
			Farm Plastic Film usage	Per ton
	Energy input		Agricultural electricity consumption	Hours per kilowatt hour
	Water resource		Agricultural irrigated area	Hundred million cubic meters
Output indicators	Desirable output		The gross output value of agriculture	Hundred million RMB
	Undesirable output		Agricultural carbon emissions	Per million tons

### 2.2 Undesirable by-products: Agricultural carbon emission measurement system and method

In measuring undesirable by-products, considering that agricultural production activities generate carbon emissions at multiple stages, combing agricultural production characteristics, and drawing on the common practice of previous literature [51–54], this paper focuses on agricultural carbon emission from three sources: agricultural production process, livestock, and farming. (1) carbon emissions caused by agricultural material inputs, such as fertilizers, pesticides, agricultural films, agricultural diesel, and agricultural irrigation; (2) methane (CH_4_) and nitrous oxide (N_2_O) emissions due to livestock, such as cattle, horses, pigs, and sheep; (3) carbon emissions arise from farming, such as CH_4_ gas emission from rice production. We use the product of carbon emission sources and their carbon emission factors as the measurement.


$$C=\sum C_{i}=\sum T_{i}\times \delta_{i}$$ (5)

In Eq (5), C represents total agricultural carbon emissions, *C*_*i*_ is carbon emissions from carbon source *i*. *T*_*i*_ and *δ*_*i*_ represent the quantity of carbon source *i* and its carbon emission coefficient, respectively. Referring to the studies of Oak Ridge National Laboratory (ORNL), Institute of Resources, Ecosystem and Environment of Agriculture, Nanjing Agricultural University (IREEA), and IPCC, we determined the carbon emission coefficients. Meanwhile, according to the IPCC Fourth Assessment Report, to aggregate emissions, CH_4_ and N_2_O were converted to standard carbon (C) by conversion factors of 6.82 and 181.27, respectively [55]. Carbon emission sources, emission factors, and references are shown in Table 2.

**Table 2:** Carbon emission sources, emission factors, and references.

Carbon Source	Index	Carbon emission	Reference
Fertilizer	Fertilizer application amount for the year	0.8956 kg/kg	ORNL
Agricultural machinery	Total power of agricultural machinery	0.18 kg/kW	Dubey
Agricultural pesticides	Number of Pesticides used per year	4.9341 kg/kg	ORNL
Agricultural plastic film	Amount of agricultural film applied in the current year	5.18 kg/kg	IREEA
Diesel fuel	Amount of diesel used per year	0.5927 kg/kg	IPCC
Agricultural irrigation	Effective irrigation area	20.476kg/hm^2^	Dubey
Agricultural electricity	Electricity consumption	0.929 kg/KW	Eggelston
Livestock and poultry	Number of breeding	CH_4_ emission factors of different livestock intestine and manure	Eggelston

### 2.3 Spatial autocorrelation and local autocorrelation of AGTFP

The AGTFP reflects a typical human-land relationship. The interactions and dependencies in the different spatial geographical ranges are crucial for us to explore their evolution characteristics and patterns. Based on Moran’s I and local Moran’s I indices, we analyze the global and local spatial correlation characteristics of AGTFP and their inter-annual variations in the YRD region. The global spatial autocorrelation describes the spatial characteristics of the study object in the whole region, which measures the overall spatial correlation and discrepancy between areas. The local autocorrelation analysis depicts local spatial heterogeneity characteristics, displaying spatial distribution patterns via identifying "hot spot areas" and "cold spot areas" in different spatial locations.


$$\begin{array}{l} I=\frac{n\sum_{i=1}^{n}\sum_{j=1}^{n}W_{ij}\left(x_{i}-\bar{x}\right)\left(x_{j}-\bar{x}\right)}{\sum_{i=1}^{n}\sum_{j=1}^{n}W_{ij}\sum_{i=1}^{n}{\left(x_{i}-\bar{x}\right)}^{2}}=\frac{\sum_{i=1}^{n}\sum_{j\neq 1}W_{ij}\left(x_{i}-\bar{x}\right)\left(x_{j}-\bar{x}\right)}{S^{2}\sum_{i=1}^{n}\sum_{i\neq j}^{n}W_{ij}}\\ S^{2}=\frac{1}{n}\sum_{j}{\left(x_{i}-\bar{x}\right)}^{2},\bar{x}=\frac{1}{n}\sum_{i=1}^{n}X_{i} \end{array}$$ (6)

Local Moran’s Index (LISA)

$$I_{i}=\frac{\left(x_{i}-\bar{x}\right)}{S^{2}}\underset{j}{\sum }W_{ij}\left(X_{j}-\bar{x}\right)$$ (7)

where I is Moran’s index, and I_i_ reflects the local Moran’s index. X_i_ is the observation value of region i. W_ij_ is the spatial weight matrix. Generally, the value of Moran’s I is at (-1, 1); if I’s value is less than 0, i.e, negative spatial correlation, it implies that this city is spatially different from the surrounding ones. If this value tends to -1, it reveals that the overall AGTFP in this region varies considerably. If I’s value is greater than 0 (positive spatial correlation), it indicates that the areas with higher (or lower) efficiency of AGTFP are spatially clustered; the greater the value tends to 1, the smaller the overall spatial difference.

### 2.4 Data sources

This paper selects 41 cities in the YRD region as the research sample, covering Shanghai, Jiangsu, Zhejiang, and Anhui Provinces. The establishment of the Yangtze River Delta Urban Economic Coordination Committee in 1999 strengthened regional cooperation and communication, marking this regional integration to an elevated stage at the collaborative level. Therefore, the window for our analysis is 2001–2019.

The data are derived from the *China Statistical Yearbook on Environment*, *China Agriculture Statistical Report*, *China Rural Statistical Yearbook*, *China Energy Statistical Yearbook*, *China Water Resources Bulletin*, and *Yangtze River Delta Prefectural and Municipal Statistical Yearbooks*.

## 3.Results

### 3.1 Changes and decomposition of AGTFP in the YRD region

Using the Malmquist–Luenberger model, we calculated the AGTFP of 41 cities in the YRD region from 2001 to 2019 and decomposed it into agricultural green efficiency change (AGEFFCH) and agricultural green technology changes (AGTECH). The calculation results are reported in Table 3. To grasp the characteristics of green productivity and the differences among the three provinces and one city, we focus on the development trend of AGTFP, AGEFFCH, and AGTECH in the subsequent analysis.

**Table 3:** Average value of AGTFP and its decomposition in the YRD region from 2001 to 2019.

id	Province	city	ml	AGTECH	AGEFFCH	id	Province	city	ml	AGTECH	AGEFFCH
1	Shanghai	Shanghai	1.014	1.000	1.014	22	Zhe jiang	Quzhou	1.010	0.991	1.019
2	Jiangsu	Nanjing	1.061	1.038	1.026	23		Zhoushan	1.003	1.000	1.003
3		Wuxi	1.117	1.046	1.073	24		Taizhou	1.075	1.001	1.075
4		Xuzhou	1.018	1.020	0.998	25		Lishui	1.008	1.000	1.008
5		Changzhou	1.040	1.027	1.029	26	Anhui	Hefei	1.027	1.000	1.027
6		Suzhou	1.101	1.048	1.050	27		Wuhu	1.047	1.000	1.047
7		Nantong	1.059	1.034	1.027	28		Bengbu	1.063	1.000	1.063
8		Lianyungang	0.980	1.003	0.977	29		Huainan	1.050	1.000	1.050
9		Huai’an	1.006	1.015	0.992	30		Ma’anshan	1.057	0.999	1.058
10		Yancheng	1.014	1.026	0.991	31		Huaibei	1.049	1.011	1.037
11		Yangzhou	1.043	1.022	1.024	32		Tongling	1.049	1.007	1.171
12		Zhenjiang	1.082	1.040	1.037	33		Anqing	1.049	1.000	1.040
13		Taizhou	1.031	1.007	1.026	34		Huangshan	1.049	1.000	1.018
14		Suqian	0.996	1.008	0.989	35		Chuzhou	1.049	0.997	1.040
15	Zhe jiang	Hangzhou	1.069	1.003	1.066	36		Fuyan	1.049	1.000	1.025
16		Ningbo	1.067	1.000	1.067	37		Suzhou	1.049	1.000	1.012
17		Wenzhou	1.032	0.979	1.055	38		Lu’an	1.049	1.000	1.019
18		Jiaxing	1.044	1.007	1.037	39		Bozhou	1.049	0.998	1.054
19		Huzhou	1.050	0.990	1.063	40		Chizhou	1.049	1.001	1.030
20		Shaoxing	1.072	1.000	1.072	41		Xuancheng	1.049	1.000	1.038
21		Jinhua	1.024	0.988	1.037						

Table 3 illustrates the average value of AGTFP and its decomposition terms in the YRD region from 2001 to 2019. It presents that the ML indices are basically greater than 1, and AGTFP shows an upward trend. From the decomposition term, the differences between the AGTFP and the AGTECH are not significant, which reveals that the sustainable growth of the AGTFP is mainly driven by the AGTECH, which is in line with the conclusion of most scholars [56,57].

Table 3 also demonstrates city differences in AGTFP in the YRD region. It displays that the deterioration of AGEFFCH is relatively obvious in the cities of Jiangsu Province, and it has little effect on the improvement of AGTFP, indicating that the business management mode of agricultural enterprises in this area needs improvement to enhance technical efficiency. The AGTFP of Wuxi and Suzhou City grew faster, and the power sources are more balanced; the AGEFFCH in these regions is higher than the AGTECH. In Zhejiang and Anhui Provinces, the growth rate of AGTFP is greater than 1, which is mainly driven by the improvement of AGEFFCH. Moreover, the value of AGEFFCH in Lianyungang city of Zhejiang Province and Suqian city of Anhui Province is lower than 1, and so do the geometric mean of this index of these two cities.

**Fig 1:**
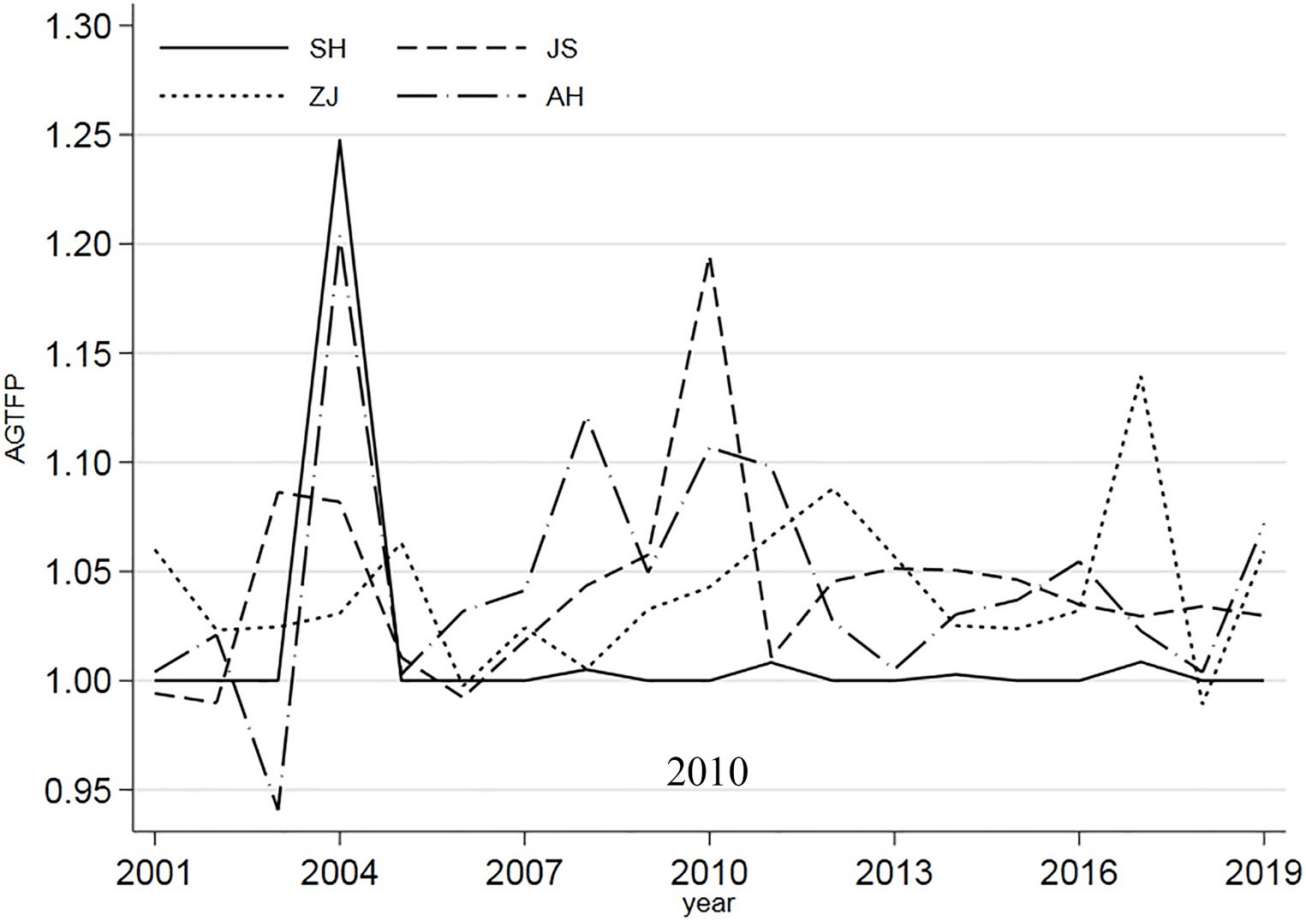
AGTFP index in the YRD region from 2001 to 2019.

Fig 1 presents the time-series changes in AGTFP in three provinces and one city in the YRD region. It shows that during the period 2001–2019, almost all provinces’ AGTFP continued to grow except for Anhui Province, whose value was below 1 in 2003, which may be due to the gradual strengthening of national environmental regulations related to green agriculture; another reason may be the different attitudes of local governments to rural ecological issues. In general, the AGTFP in Shanghai is relatively stable, while this index fluctuates more in the other three provinces, showing a U-shaped downward trend.

### 3.2 Spatial and temporal evolutionary characteristics of AGTFP in the YRD region

#### 3.2.1 Spatial evolutionary characteristics of AGTFP

**Fig 2:**
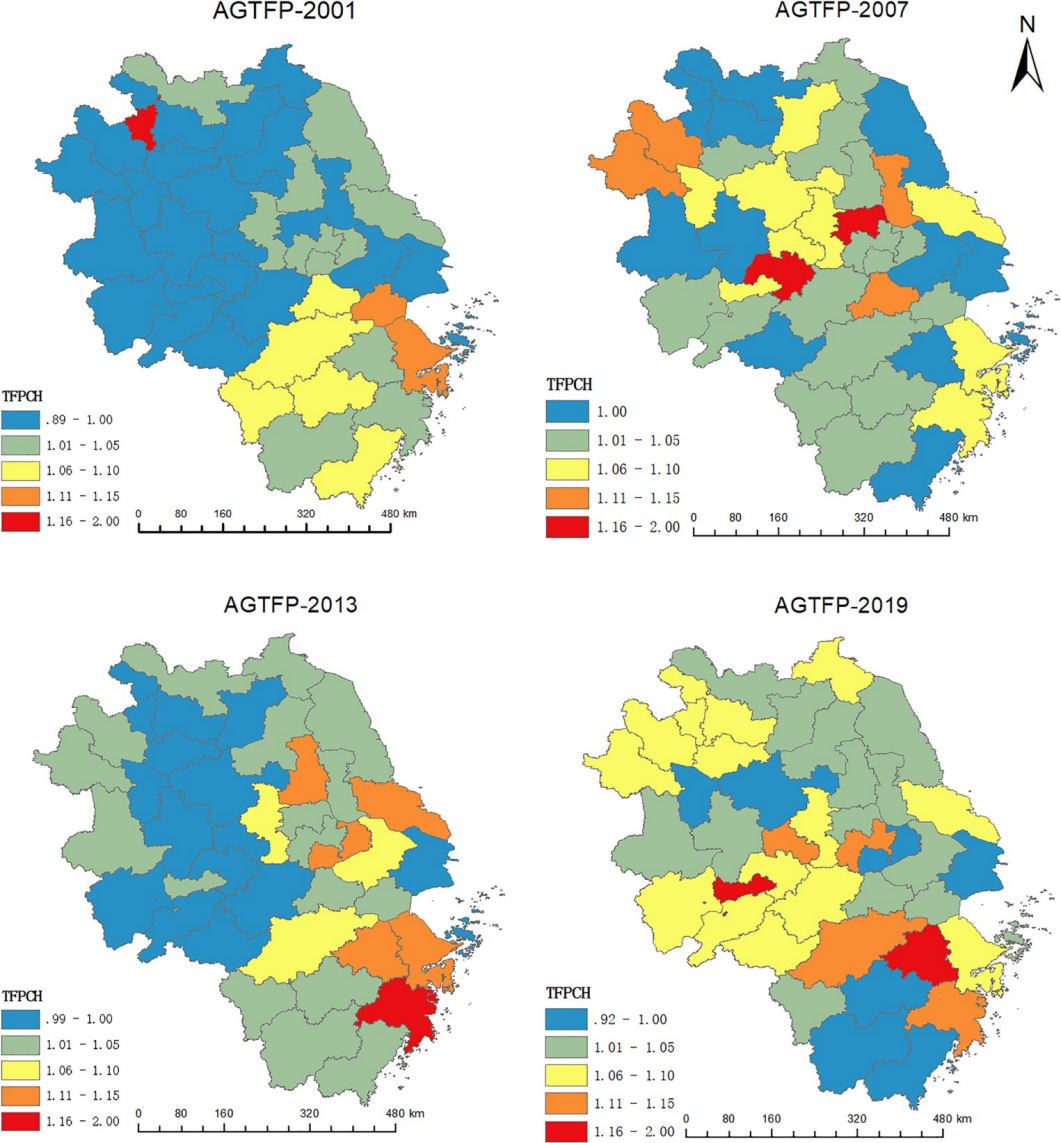
Spatial evolutionary characteristics of AGTFP (Quoted from the Tianditu map. Source of base map: The open source map data service provided by the National Platform for Common GeoSpatial Information Services) (https://www.tianditu.gov.cn).

Four years, 2001, 2007, 2013, and 2019, were selected as representative years. Adopting ArcGIS software, we visually present the spatial evolutionary characteristics of AGTFP in the YRD region (Fig 2).

Fig 2 shows two characteristics of AGTFP in the YRD region. First, there are large differences in AGTFP across cities in the same year; second, even the AGTFP of the same city varies dramatically from year to year. Compared with Fig 1A–1D reveals that cities located in the southeastern YRD region, such as Ningbo, Shaoxing, Hangzhou, and Taizhou, show a stable change in AGTFP, with high ranks. Anhui Province, located in the northwest of the YRD region, has recently seen a significant increase in AGTFP. However, the AGTFP of Shanghai is relatively low. The reason may be that Shanghai, as an industrial-oriented city, has seen its agricultural output value shrink gradually since its agricultural land has been converted to industrial land, and agricultural demand largely relies on support from neighboring provinces. Overall, the AGTFP in the YRD region shows a gradually decreasing trend from south to north.

#### 3.2.2 The test of global autocorrelation and local autocorrelation

From the initial "Shanghai Economic Zone" to the current "Integrated Development of the YRD region", regional economic activities have evolved in time and space. The spatial correlation of the AGTFP in the YRD region has gradually increased. Table 4 displays that most of the AGTFP values pass the test of Moran’s I index, and it can be concluded that AGTFP has spatial correlation. Although the global Moran’s I index of AGTFP fluctuates slightly, the overall trend is increasing.

**Table 4:** Results of the global spatial correlation test.

year	I	P- value	Year	I	P- value
2001	0.1756	0.0209	2011	0.2413	0.0014
2002	0.0342	0.5109	2012	0.3573	0.0001
2003	0.1945	0.001	2013	0.2923	0.0007
2004	0.1486	0.0506	2014	-0.1507	0.193
2005	0.1965	0.0091	2015	-0.1526	0.1646
2006	-0.0559	0.6772	2016	-0.1156	0.304
2007	0.1131	0.1294	2017	0.374	0
2008	0.1978	0.0142	2018	0.0309	0.5498
2009	0.0474	0.4189	2019	0.095	0.1738
2010	0.0256	0.5637			

Although Table 4 demonstrates that the AGTFP of cities in the YRD region has a global spatial correlation, the index cannot show which regions agglomerate and cannot portray their spatial autocorrelation relationship; therefore, we need to further examine the local spatial autocorrelation of AGTFP.

**Fig 3:**
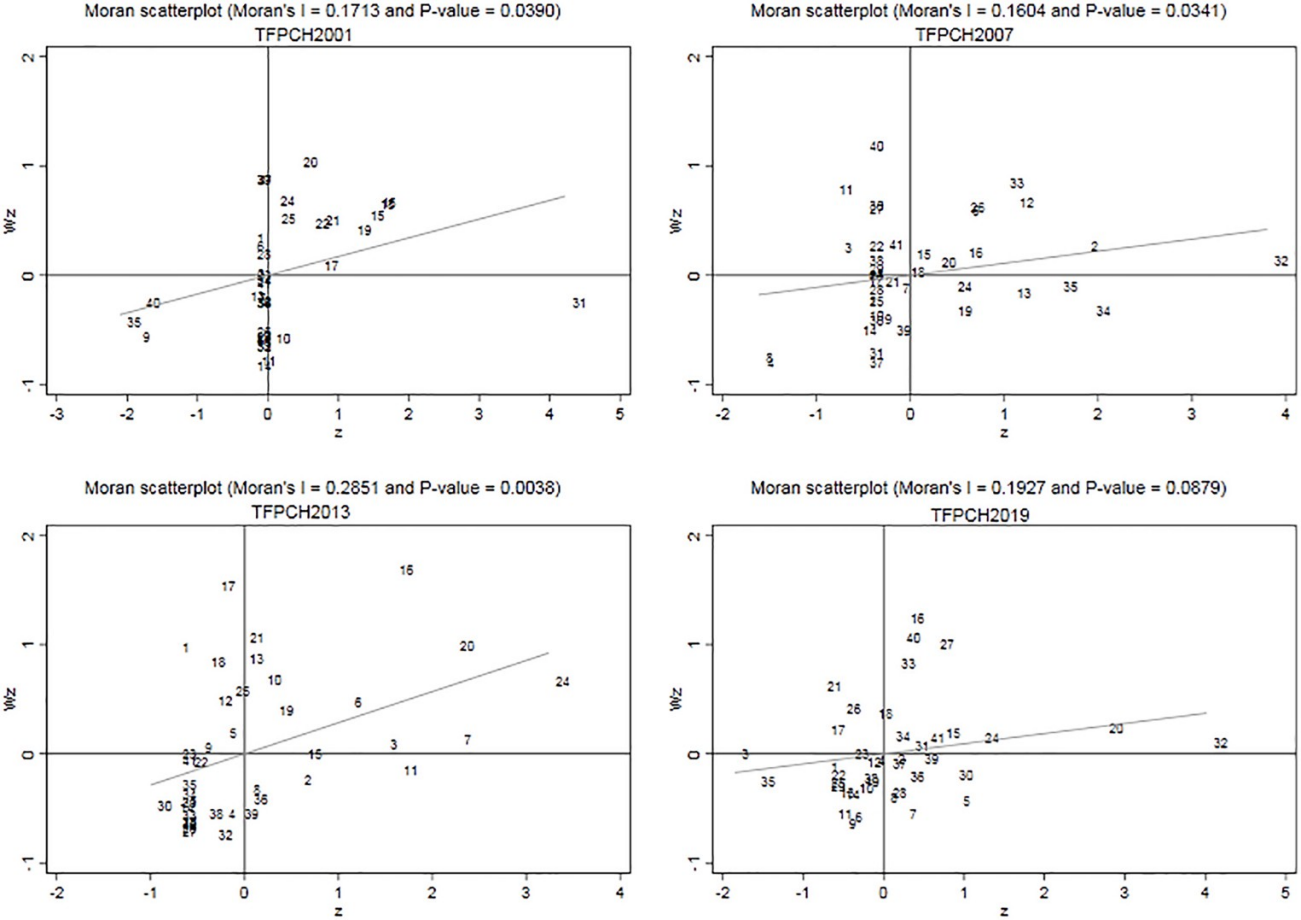
Scatter plot of Moran’s I index.

The scatter plot of the local Moran’s I index reflects spatial agglomeration. We use 2001, 2007, 2013, and 2019 as the representative years. Fig 3 shows the scatter plot of the local Moran’s I index. The x-coordinate is z, which represents standardized observations of the spatial unit; the y-coordinate is Wz, indicating the average value of standardized observations of adjacent units. The slope of the correlation curves presents that the spatial correlation of AGTFP is significantly lower in 2019 than in 2001 and 2013. The numbers 1–41 in the four quadrants of Fig 3 represent cities, and Tables 5 and 6 show the specific city names and quadrant distributions.

**Table 5:** Quadrant distribution of cities in the YRD region in 2001.

Quadrant	Quadrant nature	City
First quadrant	High—High	Hangzhou,Wenzhou, Ningbo, Huzhou, Jiaxing, Shaoxing, Jinhua, Lishui, Taizhou, and Quzhou
Second quadrant	Low—High	Shanghai, Wuxi, Xuzhou, Suzhou, Bengbu, Suizhou, and Bozhou
Third quadrant	Low—Low	Nanjing, Nantong, Changzhou, Huaian, Lianyungang, Yangzhou,Taizhou, Hefei, Zhenjiang, Suqian, Wuhu, Huainan, Tongling, Maanshan, Anqing, Chuzhou, Huangshan, Fuyang, Chizhou, Xuancheng, and Liuan
Fourth quadrant	High—Low	Yancheng and Huaibei
The junction of the first and fourth quadrants		Zhoushan

**Table 6:** Quadrant distribution of cities in the YRD region in 2019.

Quadrant	Quadrant nature	City
First quadrant	High—High	Hangzhou, Ningbo, Shaoxing, Taizhou, Wuhu, Huaibei, Tongling, Anqing, Huangshan, Chizhou, and Xuancheng
Second quadrant	Low—High	Wuxi, Wenzhou, Jiaxing, Jinhua, and Hefei
Third quadrant	Low—Low	Shanghai, Xuzhou, Suzhou, Lianyungang, Huaian, Yancheng, Yangzhou, Zhenjiang, Taizhou, Suqian, Huzhou, Quzhou, Lishui, Huainan, Chuzhou, and Liuan
Fourth quadrant	High—Low	Nanjing, Changzhou, Nantong, Bengbu, Maanshan, Fuyang, Suizhou, and Bozhou
The junction of the first and fourth quadrants		Zhoushan

Note: "High—High" indicates that cities with high AGTFP are surrounded by cities with high AGTFP. "Low—Low" implies that cities with low AGTFP are surrounded by cities with low AGTFP. "High—Low" means that cities with high AGTFP are surrounded by cities with low AGTFP.

We can see the findings from the four-year city quadrant distribution.

1. The cities in the first quadrant are Wuxi, Hangzhou, Ningbo, Shaoxing, and Taizhou. The AGTFP of these cities is high, and so do their neighboring cities. No cities in Jiangsu Province are located in the first quadrant.
2. The cities in the third quadrant are those in Anhui Province and those in the western and northern parts of Jiangsu Province. The AGTFP of these cities and their neighboring cities is low. No cities in Zhejiang Province entered the third quadrant.
3. By comparing the quadrant changes, we can see that the cities with faster AGTFP improvement are Wuhu, Huaibei, Tongling, Anqing, Huangshan, Chizhou, Xuancheng, etc.

In general, most cities in Zhejiang Province have higher AGTFP, while most cities in Anhui Province have lower AGTFP. The AGTFP in the eastern cities of Jiangsu Province is higher than that in the western and northern cities. The cities of Shanghai and Nanjing had high AGTFP in the early stage but declined faster in the later stage, which may be due to urbanization and industrialization, where agricultural land is converted into industrial land, limiting agricultural development. Cities in western Anhui Province, such as Tongling, Anqing, Huangshan, and Chizhou, have dramatically improved their AGTFP.

## 4. Convergence analysis

The neoclassical growth model assumes that the final state of economic development tends to be stable, i.e., there is bound to be convergence. To achieve the goal of green growth, we need to analyze the internal development gaps in the YRD region. The above has confirmed that there are regional differences in the AGTFP in this region; however, such inter-regional differences could vary with time. Therefore, to clarify the changes in the AGTFP gap, we need to calculate its convergence. The literature measures convergence mainly by calculating α-convergence and β-convergence; since the two calculation methods focus on different points, the results are quite different.

### 4.1 α convergence analysis

α convergence could present the variation in AGTFP with time in the YRD region and reflect the degree of dispersion. If this index declines, it indicates that AGTFP in the YRD region converges; otherwise, it diverges. Based on the study of Zhao [58], we adopt the standard deviation to measure whether there is a convergence of AGTFP in this region.


$$\sigma_{t}=\sqrt{\frac{1}{t-1}\sum_{i=1}^{n}{\left(ML_{i,t}-\bar{ML}\right)}^{2}}$$ (8)

**Fig 4:**
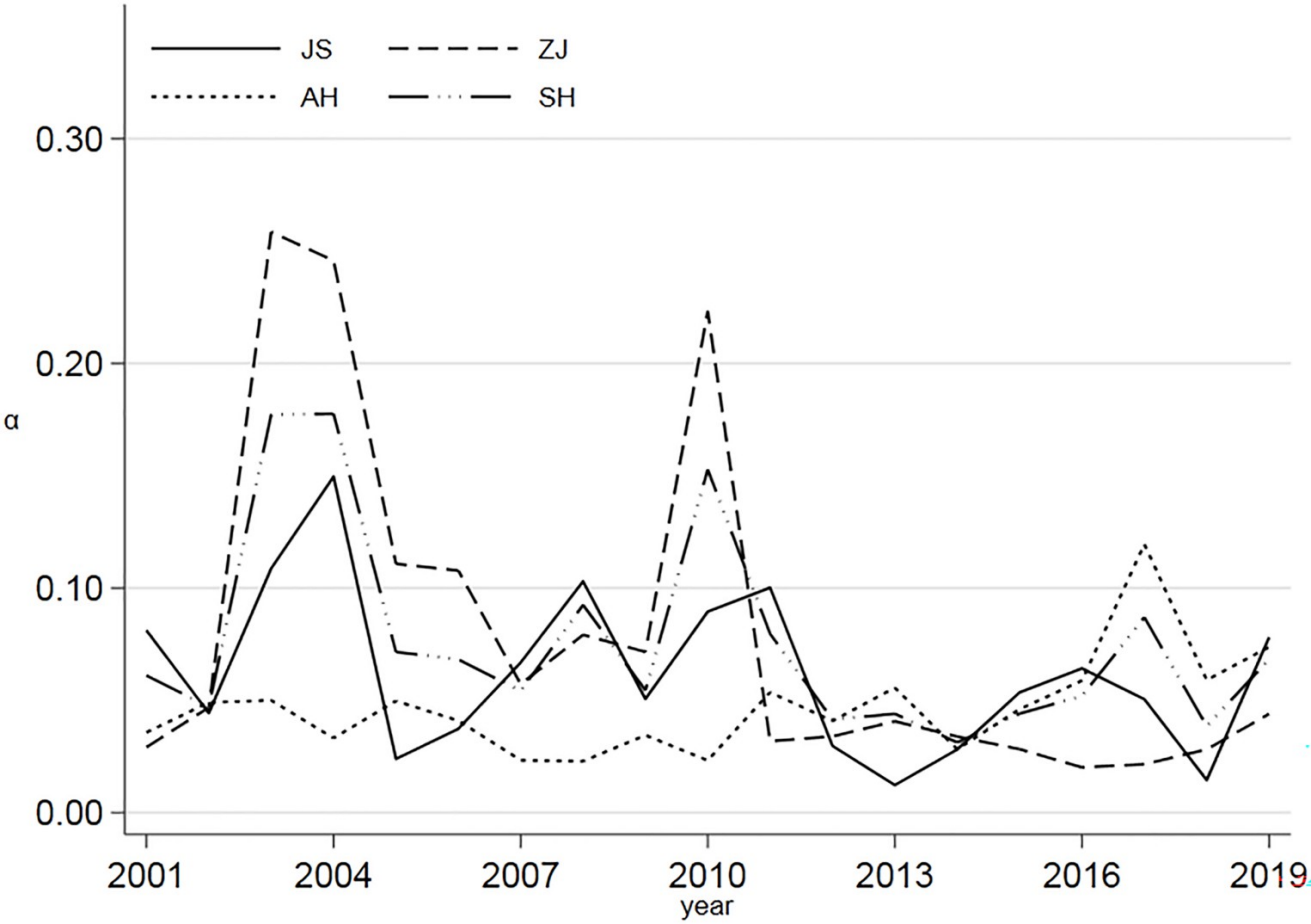
The trend of α convergence of provincial AGTFP in the YRD region.

Fig 4 shows that the AGTFP in this region does not show a decreasing trend over time. In contrast, the α convergence gradually increases with time, displaying a "W"-shaped distribution, and after 2018, it increases with the year. Therefore, there is no significant α convergence in AGTFP in the YRD region.

Moreover, Fig 3 shows that comparing the AGTFP in the east and west of the YRD region, there is no α convergence. The α values of Jiangsu, Zhejiang, Anhui, and Shanghai all fluctuate greatly, indicating that these regions’ AGTFP is not stable. This instability reveals that the perceptions of green growth and transformation are insufficient. Despite the differences in the foundation and resource environment, development speed, and scale of the YRD region, such differences still exist and have not been able to stabilize over time during the sample period.

### 4.2 β convergence analysis

Absolute *β* convergence mainly investigates whether a variable has the same development trend in a certain period and whether it will converge to the final steady state. We use absolute *β* convergence to test whether the AGTFP in the YRD region will converge to the same steady state. If the AGTFP of 41 cities in the YRD region shows variability, this variation may be due to resource endowments or accumulated institutional issues. If absolute *β*-convergence of AGTFP exists in the YRD region, it means that cities with low AGTFP are catching up with cities with high AGTFP. This "catch-up effect" implies that the two will converge to the same steady state. Based on Barro et al. [59], we measure the absolute *β* convergence of AGTFP in the YRD region, and the spatial test model is as follows.

$$\ln(\frac{TFP_{i,t}}{TFP_{i,0}})=\alpha +\beta \ln TFP_{i,0}+\varepsilon_{i}$$ (9)

$$\ln(\frac{TFP_{i,t}}{TFP_{i,0}})=\alpha +\beta \ln TFP_{i,0}+\lambda W_{i}\ln(TFP_{i,t-1})+\varepsilon_{i}$$ (10)

where *λ* is the spatial autoregressive coefficient and *W*_*i*_ denotes the row *i* of the spatial weight matrix. Compared with the absolute convergence model (9), model (10) introduces the spatially lagged term of AGTFP in neighboring regions to illustrate the correlation between the AGTFP of one region and its neighboring regions.

To enhance the robustness of the results, we use the adjacency matrix to construct the spatial weight matrix. The Hausman test results support the fixed effects model. The results of Wald and LR tests reveal that the dynamic spatial Durbin model with Spatio-temporal lagged terms of the explanatory variables can be judged as an optimal estimation model for the measure of spatial β convergence. Table 7 shows that among the three spatial econometric models of absolute β convergence, the results of the spatial lagged model (SLM), spatial error model (SEM), and spatial Durbin model (SDM) are all robust, and spatial effect coefficients λ or ρ are significantly greater than 0, implying that cities’ AGTFP in the YRD region has a significant spatial spillover effect.

**Table 7:** Results of the spatial convergence model of AGTFP in the YRD region.

Variables	Absolute β convergence	Spatial absolute β convergence		
		SLM	SEM	SDM
LTFPCH	-0.986***	-0.967***	-0.911***	-0.961***
	(0.03831)	(0.0367)	(0.0362)	(0.0372)
λ		0.417***		
		(0.0433)		
ρ			0.295***	0.414***
			(0.0364)	(0.0434)
Sigma^2^		0.00726***	0.00760***	0.00726***
		(0.000384)	(0.000397)	(0.000383)
w LTFPCH				0.344***
				(0.0775)
Astringent	convergence	Convergence	convergence	convergence
Convergence speed	0.0141	0.0336	0.0932	0.0399
N	738	738	738	738
R^2^	0.571	0.431	0.298	0.416

Note: Standard errors are in parentheses

* p < 0.1

** p < 0.05

*** p < 0.01.

The convergence characteristics can be obtained from the regression results. (1) There is a significant convergence trend of AGTFP in the YRD region. The AGTFP of all convergence models is significantly negative, and the estimated coefficients of the differences in AGTFP are decreasing across regions. It means that cities with low AGTFP are catching up with cities with high AGTFP in this region. The results of the SDM show that the spatial spillover effect of AGTFP is positive. The convergence rates of AGTFP under different spatial models are 3.36%, 9.32%, and 3.99%, respectively. (2) The absolute β convergence with the addition of spatial factors further accelerates the convergence rate of AGTFP more than the absolute β convergence, probably because the spatial spillover effect reinforces the interaction between neighboring regions, especially the spatial transfer and sharing of agricultural green technical efficiency and technological progress between regions facilitate the spatial spillover effects of AGTFP. Therefore, the spatial discrepancies of AGTFP in the YRD region tend to narrow with time, and the convergence rate has accelerated.

## 5. Discussion

The GTFP provides decision information for land planning in the YRD region. Extensive land management and over-exploitation may cause land fertility decline, leading to soil pollution and environmental damage [60,61]. Technological progress in AGTFP in the YRD region should focus on providing resource-saving and labor-saving technologies for land use, which can provide support for improving land use. Therefore, the goals of saving land, reducing land use, and improving land efficiency could be achieved. Moreover, technological progress helps to increase land protection, especially for arable land. Arable land protection and arable land planning should be incorporated into the goals of ecological priority and green development, and the ecological function of arable land, that is, the virtuous cycle of the ecosystem, should be fulfilled.

The AGTFP offers guidance for the layout of land planning in the YRD region. The improvement of technological efficiency reflects the continuous approach of production units to the boundary of production possibilities. The objects of agricultural labor are living plants and animals; the transfer of agricultural technology requires adaptation to natural conditions and improving infrastructure. Specifically, relying on rich water resources, the YRD region could make planning for green agricultural demonstration zones, vigorously develop recycling, ecological, and leisure agriculture, and promote the integration of the three industries. Meanwhile, based on technical efficiency evaluation, the YRD region could optimize the layout of village land. Moreover, this region should encourage the compound use of land for agricultural production and village construction, and promote the deep integration of agriculture with tourism, culture, education, recreation and other industries.

The AGTFP provides a theoretical basis for regional coordinated development. AGTFP is conducive to discerning the level of land ecological management, which provides a reference for improving various spatial layouts under the premise of total arable land balance, quality improvement, and structural optimization [62]. In particular, the adjustment of the basic farmland layout within the urban development boundary and ecological protection red line in the demonstration area of the YRD region.

## 6. Conclusions and recommendations

### 6.1 Conclusions

Based on the theory of sustainable development, from the dimension of desirable and undesirable output, we construct an ecological environment evaluation system and incorporate it into the framework of AGTFP; we measure AGTFP from 2001 to 2019 using a two-period Malmquist–Luenberger index under a carbon emission constraint. Furthermore, we analyzed the global spatial correlation and local spatial correlation of AGTFP in the YRD region using the Moran index and scatter plot method. Moreover, we depict and explain regional differences and spatial convergence of AGTFP in the YRD region. We find that:

First, the growth of AGTFP in the YRD region is propelled by technical efficiency, while technical progress is insufficient, and its growth rate shows a "U" shaped downward fluctuation trend, which is the main reason for the fluctuation of AGTFP in the YRD region. Two aspects of the problem emerge under the requirements of regional integration. Specifically, on the one hand, the growth of AGTFP of cities in the YRD region has not yet achieved inter-regional coordinated development; on the other hand, there are small fluctuations in the growth of cities’ AGTFP in this region, with the fluctuation of "growth-decline-growth".

Second, overall regional differences in AGTFP in the YRD region continually widen, and intra-regional imbalance also emerges. The inter-regional imbalance is the main reason for the overall regional differences in AGTFP. The contributions of EFFCH and TECH to the growth of AGTFP show obvious differences. The agricultural green development in the YRD region has not yet achieved the synergistic growth of technical efficiency improvement and technological progress, and technological progress still restricts the sustainable growth of cities’ AGTFP in the YRD region.

Third, there is an obvious global spatial agglomeration of AGTFP in the YRD region, and the agglomeration wanes with time. β convergence of AGTFP in the YRD region passes the significance test, indicating that the growth rate of AGTFP in this region begins to converge. Furthermore, cities’ AGTFP in this region exhibits positive spatial spillover effects.

### 6.2 Recommendations and implications

Improving AGTFP is crucial for enhancing rural revitalization and achieving high-quality agricultural development. It is the key initiative to transform the development mode, improve economic efficiency, and convert the growth momentum of agriculture. It is also an important policy focus point for realizing common prosperity in rural areas and the overall coordinated development of agriculture in the YRD region. Based on the results of regional differences and spatial convergence of AGTFP in the YRD region, we propose the following recommendations.

Firstly, we should enhance technological progress. On the one hand, we should increase the introduction and research, and development of advanced agricultural technologies, actively expand the opening of foreign technologies, promote the abundance of agricultural human capital and agricultural innovation and entrepreneurial resources, and thus act as the driving force of technological progress on the enhancement of AGTFP; on the other hand, we should accelerate the upgrading of agricultural industries, enhance the effective use of clean energy, green production, and green agricultural management models, and deeply improve green agricultural development.

Secondly, we should coordinate the development of the agricultural economy in the YRD region. Specifically, on the one hand, we should deepen the planning of regional agricultural development, promote the demonstration role of cities with better agricultural development, realize the effective concentration of green agricultural technology and innovative resources, and take advantage of the scale effect; on the other hand, we should improve the agricultural industrial structure, strengthen the construction of agricultural economic belts and agricultural economic circles, optimize the regional agricultural spatial layout, and improve the usage efficiency of agricultural resources.

Finally, we should strengthen the influence of the institutional system in the YRD region on the AGTFP. Specifically, we should build a synergistic mechanism of regional financial and environmental protection systems, promote regional synergistic environmental management of agricultural production, improve the carbon sink compensation mechanism between regions, give full play to regional comparative advantages, cultivate agricultural staple industries suitable for cities in the YRD region according to local conditions, and thus promote cities’ AGTFP in the YRD region.

## Supporting information

S1 File AGTPF data of 41 cities in the Yangtze River Delta Region from 2000 to 2020. AGTPF calculation results of 41 cities including values of AGTFP, AGEFFCH and AGTECH by using the Malmquist-Luenberger model. These data were used to generate Tables 3–7 and Figs 1–4.
(RAR)

S2 File AGTFP index value in the Yangtze River Delta Region from 2000 to 2019. AGTPF raw data of 16 cities in Anhui, 13 cities in Jiangsu, Shanghai and 11 cities in Zhejiang from 2000 to 2019. These data were used to generate Figs 1 and 4.
(RAR)
